# Dietary shifts and gut microbiota plasticity correlates of habitat micromodification in wild oriental storks: implications for conservation physiology

**DOI:** 10.3389/fvets.2026.1769005

**Published:** 2026-02-10

**Authors:** Yifan Zhou, Menglin Sun, Zeming Zhang, Hong Wu, Dapeng Zhao

**Affiliations:** Tianjin Key Laboratory of Conservation and Utilization of Animal Diversity, College of Life Sciences, Tianjin Normal University, Tianjin, China

**Keywords:** adjustments adaptation, diet, gut microbiota, habitat micromodification, oriental storks

## Abstract

Understanding how wetland habitat micromodification impacts the health of birds through dietary and microbial adjustments is critical for their conservation. Tianjin Qilihai Wetland serves as a critical migration stopover site for the oriental stork (*Ciconia boyciana*), while having undergone a habitat micromodification. In this study, the fecal samples of oriental storks across pre-change group (collected in 2022), under-change group (collected in 2023), and post-change group (collected in 2024) from Tianjin Qilihai Wetland were analyzed by integrating fecal microhistology with 16S rRNA sequencing. The results showed that, at the phylum level, the dominant bacterial phyla of oriental storks in the three years all contained Firmicutes and Actinobacteria, and at the genus level, the dominant bacterial genera of gut microbiota were *Paeniclostridium* and *Lactobacillus*. The abundances of *Paeniclostridium* and *Lactobacillus* were highest in under-change group. Ten species of plants belonging to 10 genera and 8 families were identified based on microscopic examination, of which *Abutilon theophrasti* was unique in pre-change group, *Suaeda glauca* and *Nelumbo nucifera* were unique in under-change group. During the environmental micromodifications, the quality of the wetland environment declined, and the types and quantity of food resources available changed, which in turn affected the diet choice and gut microbiota structure of oriental storks. The research provides a reference for wetland micromodifications and wildlife conservation.

## 1 Introduction

Habitat stability has a significant impact on maintaining the life activities of species. The environmental quality directly impacts migration behavior, reproductive success, foraging efficiency (1–3), and threatens population health and dynamics (4, 5). Bird migration exemplifies this complexity, requiring multiple contributing factors (6, 7), where stopover habitat alterations represent significant influences. The avian gut microbiota plays a fundamental role in maintaining host health and homeostasis (8, 9). This complex ecosystem regulates nutrient absorption, immune function, and pathogen defense (10, 11). Wild birds demonstrate microbiome plasticity through dietary, developmental, and environmental adaptations (12–15), suggesting particular importance for migratory species facing diverse ecological challenges, therefore the significance of related research on conservation physiology is emphasized (16).

The oriental stork (*Ciconia boyciana*) belongs to the genus *Ciconia* of the family Ciconiidae under the order Ciconiiformes, is a large wading bird rated as endangered (EN) in the IUCN red list (17). Current research focuses on population dynamics, diet, gut microbiota, behavior, and habitat selection (18–22), though comprehensive studies remain limited. Naito (23) and Tawa (19) found that oriental storks were carnivorous birds, mainly feeding on fish, amphibians, crustaceans, insects, etc. However, the oriental stork also fed on plant-based parts (24), it is necessary to consider the protection of plant resources in their habitats.

Diet-microbiota relationships are established in avian species. Wu et al. (25) demonstrated that the consumption of *Triticum aestivum*, *Potentilla chinensis*, and *Zea mays* influenced the abundance of Firmicutes in the gut microbiota of *Grus grus*. Wang et al. (26) reported elevated Clostridiaceae levels in *Grus leucogeranus* foraging on Vallisneria tubers compared to other dietary groups. Similarly, Bodawatta et al. (27) observed adaptive microbial shifts in *Parus major,* where exclusive mealworm feeding triggered increased abundances of Lactobacillales, Bacillales, and Clostridiales. However, the mechanistic links between dietary patterns and gut microbiome structure remain unknown in oriental storks, particularly regarding how specific nutrients drive microbial functional adaptations.

Habitat micromodification can improve the suitability of species, enrich the food resources of species, and improve the success rate of species reproduction through wetland hydrological regulation, vegetation community optimization, and the establishment of artificial nests (28–30), which is one of the effective means to protect the diversity of wild animals. However, habitat micromodification may trigger trophic cascades that alter food web dynamics. During the transformation period, it may lead to the decline of benthic animal biomass, fluctuations in fish resources, etc. (31, 32). These changes will force the adaptive transfer of species’ feeding strategies. For example, Hebert et al. (33) pointed out that *Larus argentatus* increased consumption of lower-trophic-level prey when fish resources decline, while *Leucogeranus leucogeranus* exploited food resources in artificial habitats to meet energy demands when natural food availability decreased (34). Food changes will affect the richness and diversity of gut microbiota. Understanding the changes of species’ food composition and gut microbiota composition before and after habitat micromodification is conducive to monitoring the effectiveness of environmental transformation and providing basis for habitat micromodification. During the period from 2022 to 2024, the management department of Tianjin Qilihai Wetland carried out habitat micromodification on the wetland environment, providing an ideal external condition for investigating the comprehensive relationship between the diet and gut microbiota of oriental storks during the habitat micromodification period. This study investigated the gut microbiota and diet of the oriental stork using non-invasive techniques, in order to understand how environmental micromodification affects the health status and diet composition of wild oriental storks.

## 2 Materials and methods

### 2.1 Sample collection

Fecal samples were collected from wild oriental storks in Tianjin Qilihai Wetland (39°16′N-39°19’N, 117°27′E-117°38′E), which is located in the migratory flight route of East Asia-Australasia migratory birds and rich in wildlife resources, so it is an important resting and migrating place for oriental storks. Within the Tianjin Qilihai Wetland, the work of habitat micromodification that include dredging of river channels and reinforcing of corresponding embankments across some water areas and were carried out in 2023. This study collected samples across three time periods including Pre-change group (2022, 10), Under-change group (2023, 10), and Post-change group (2024, 8) (Supplementary Table S1).

Fecal sample collection were performed only on oriental storks that were continuously observed in Tianjin Qilihai Wetland for no less than 7 days. A total of 28 fecal samples were collected immediately after confirming the excretion behavior and stored in 5 mL sterile EP tubes. Then, samples were stored at −80 °C until further processing. After sample collection, it was essential to document habitat characteristics of the sampling sites and collect/identify plant species distributed within the immediate vicinity of the samples.

### 2.2 DNA extraction and sequencing

The TIANamp Stool DNA Kit (TIANGEN, China) was used to extract genomic DNA from fecal samples. After extraction, the Nano Drop 2000 spectrophotometer (Thermo Fisher Scientific, United States) was used to determine the concentration and quality of DNA. The V3–V4 hypervariable regions of the 16S rRNA gene were amplified using universal bacterial primers (338F 5’-ACTCCTACGGGA GGCAGCA-3′ and 806R 5’-GGACTACHVGGGTWTCTAAT-3′). The PCR reaction was prepared in a 20 μL mixture containing 4.0 μL of 5 × Fast Pfu Buffer, 2.0 μL of 2.5 mmol/L dNTPs, 0.8 μL each of forward and reverse primers (1 μmol/L), 0.4 μL Fast Pfu DNA Polymerase, 0.2 μL BSA, and 10 ng template DNA, with the final volume adjusted to 20 μL using ddH₂O. PCR amplification was performed under the following conditions: initial denaturation (95 °C, 3 min); 27 cycles of denaturation (95 °C, 30 s), annealing (55 °C, 30 s), and extension (72 °C, 45 s); final extension (72 °C, 10 min). The purified PCR amplicons were sequenced on the Illumina MiSeq platform by Majorbio Bio-pharm Technology Co., Ltd. (Shanghai, China).

Raw paired-end sequencing data underwent quality control using Fastp, followed by sequence assembly with FLASH v1.2.11.^1^ The workflow implemented: Sliding-window scanning (10-bp window) starting from the 3′-end of reads, truncating subsequent bases when the average Q-score within the window fell below 20; Removal of reads shorter than 50 bp post-truncation and those containing ambiguous bases (N); Assembly with a maximum overlap mismatch rate threshold of 0.2.

### 2.3 Bioinformatics analysis

Raw sequencing data were processed using QIIME2 (35). After quality filtering and denoising, sequences were clustered into Operational Taxonomic Units (OTUs) at 97% similarity using the UPARSE algorithm (36). Taxonomy was assigned using the SILVA database.

Diversity and statistical analyses Alpha diversity indices (Ace, Chao, and Shannon) were calculated to assess gut microbiota richness and diversity. Beta diversity was evaluated using unweighted and weighted uniFrac distances, visualized through Principal Coordinate Analysis (PCoA). Kruskal-Wallis rank-sum tests were used for multi-group comparisons, while Wilcoxon rank-sum tests were used for pairwise comparisons. PICRUSt2 was utilized to predict the functional potential of the microbial communities based on 16S rRNA gene sequences (37). The resulting functional profiles were analyzed at the KEGG pathway level (38). BugBase was employed to predict the potential pathogenicity of the gut microbiota and analyze species contributions to pathogenicity (39). All statistical analyses and visualizations were performed using *R* (version 4.0.0) with relevant packages (e.g., vegan, ggplot2).

### 2.4 Food analysis

The collected plant and feces samples of oriental storks were pretreated according to the method from Mo et al. (40). When treating plants, different parts of the plants (such as flowers, seeds, and stems) should be pretreated, respectively, and the plant cells were magnified 100 times to record the morphology by microscope. When examining fecal samples, four slides needed to be prepared. After magnifying 100 times, 10 non-overlapping fields of view were counted. Based on the structure of the plant cells that had been captured, the types of plants in the fecal samples were determined. Statistically identified the species and occurrence frequency (*F*) of plants, and converted the frequency into average density (*D*):


$$D_{i}=-\ln(1-F/100)$$

Convert the average density (*D*) into the relative density (*RD*):


$$\mathrm{RD}=(D_{i}/\sum D)\times 100\%$$

*D_i_* represented the density of recognizable keratin fragments of a certain plant, and Σ*D* represented the sum of recognizable keratin fragments of all plants. The relative density was used to estimate the actual proportion of plants.

Inter-taxonomic correlations between dominant gut microbiota components (at phylum and genus levels) and dietary plant species of oriental storks were assessed using bivariate Spearman analysis in Origin 2022 (version 9.7).

## 3 Results

### 3.1 Gut microbiota diversity and function prediction analysis

#### 3.1.1 Basic sequencing information

The data of 28 samples were analyzed and denoised by Illumina MiSeq sequencing. A total of 1,650,421 optimized sequences were obtained, with an average length of 414 bp. The OTUs were classified based on 97% similarity, and a total of 6,606 OTUs were counted, including 2,747 unique OTUs for samples in the pre-change group, 106 unique OTUs for samples in the under-change group, 1,510 unique OTUs for samples in the post-change group. There were 562 OTUs shared among the 3 years (Supplementary Figure 1C).

The gut microbiota of oriental storks comprised 56 phyla, 164 classes, 407 orders, 673 families, and 1,279 genera. Specifically, pre-change group contained 53 phyla, 158 classes, 376 orders, 586 families, and 1,010 genera. Under-change group contained 27 phyla, 70 classes, 164 orders, 250 families, and 385 genera. Post-change group contained 46 phyla, 131 classes, 322 orders, 519 families, and 1,000 genera.

Pan OTU and Core OTU refer to the total OTUs and shared OTUs among samples, respectively. As the sample size increased, Pan OTU gradually increased and tended to plateau, indicating that the total OTUs of the three-year samples gradually increased (Supplementary Figure 1E). The gradual plateauing of both the Pan/Core OTU curves and Shannon index rarefaction curves indicated that the sample size in this study was sufficient, and the sequencing results basically covered the major components of microbial diversity in the samples (Supplementary Figures 1D–F).

#### 3.1.2 Phylum and genus level diversity analysis

**Figure 1:**
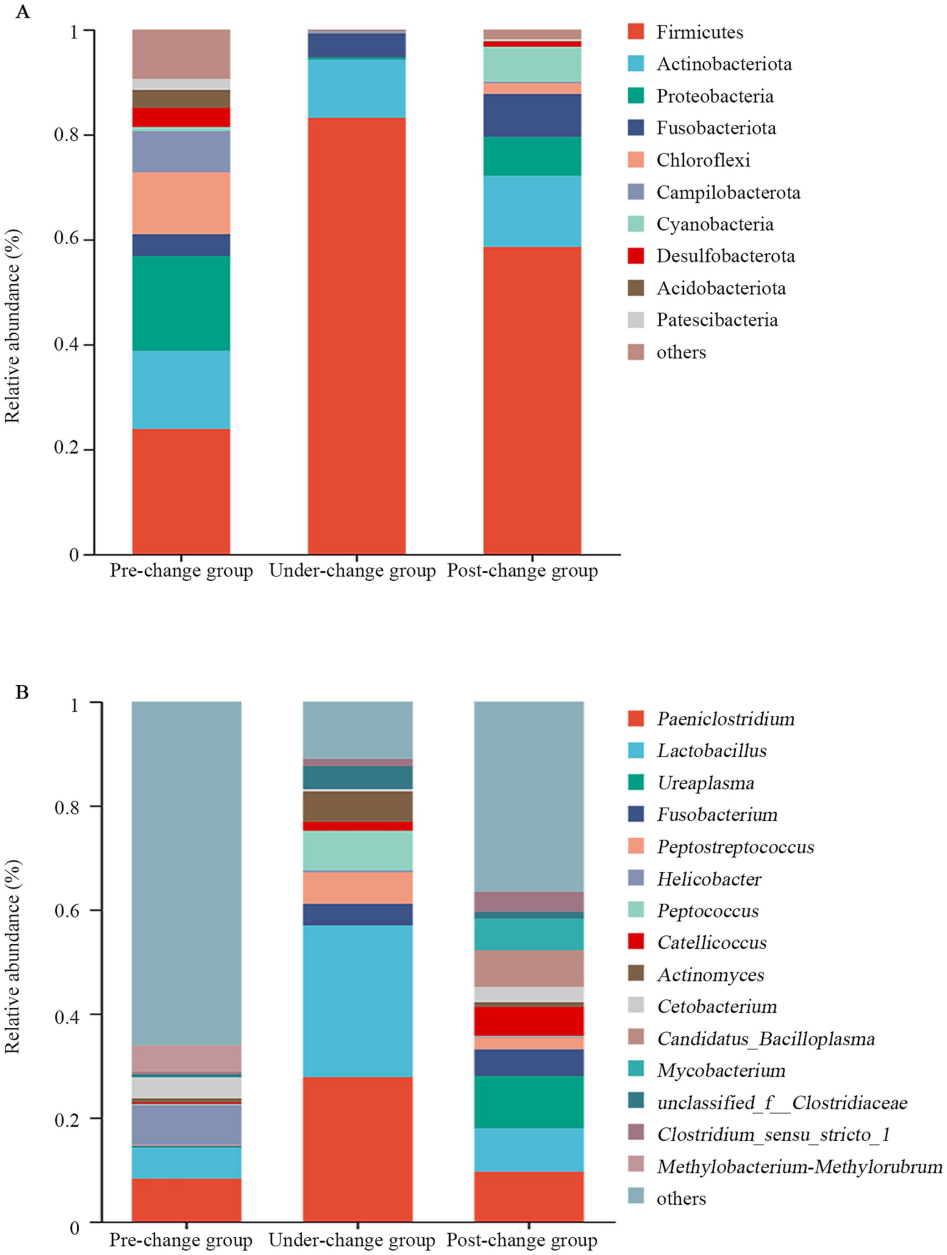
Microbial structure of all fecal samples from oriental storks at the phylum **(A)** and genus levels **(B)** across 3 years.

At the phylum level, the dominant phyla in pre-change group were Firmicutes (23.89%), Proteobacteria (18.11%), Actinobacteriota (14.87%), Chloroflexi (11.79%), and Campilobacterota (7.89%). In under-change group, the dominant phyla were Firmicutes (83.19%), Actinobacteriota (11.08%), Fusobacteriota (4.60%), Proteobacteria (0.40%), and Campilobacterota (0.39%). In post-change group, the dominant phyla were Firmicutes (58.60%), Actinobacteriota (13.54%), Fusobacteriota (8.18%), Proteobacteria (7.40%), and Cyanobacteria (6.67%). The dominant phyla in the three groups all included Firmicutes and Actinobacteriota (Figure 1A; Supplementary Table S2).

At the genus level, the dominant genera in pre-change group were *Paeniclostridium* (8.29%), *Helicobacter* (7.72%), *Lactobacillus* (6.00%), *Methylobacterium-Methylorubrum* (5.07%), and *Cetobacterium* (4.00%). In under-change group, the dominant genera were *Lactobacillus* (29.17%), *Paeniclostridium* (27.79%), *Peptococcus* (7.61%), *Peptostreptococcus* (6.05%), and *Actinomyces* (5.82%). In post-change group, the dominant genera were *Ureaplasma* (10.02%), *Paeniclostridium* (9.62%), *Lactobacillus* (8.35%), *Candidatus_Bacilloplasma* (7.05%), and *Mycobacterium* (6.08%). The dominant genera in the three groups all included *Lactobacillus* and *Paeniclostridium*. In pre-change group, the specific dominant genera were *Helicobacter, Methylobacterium-Methylorubrum,* and *Cetobacterium*. In under-change group, the specific dominant genera were *Peptococcus*, *Peptostreptococcu,* and *Actinomyces*. In post-change group, the specific dominant genera were *Ureaplasma*, *Candidatus_Bacilloplasma,* and *Mycobacterium* (Figure 1B; Supplementary Table S2).

**Figure 2:**
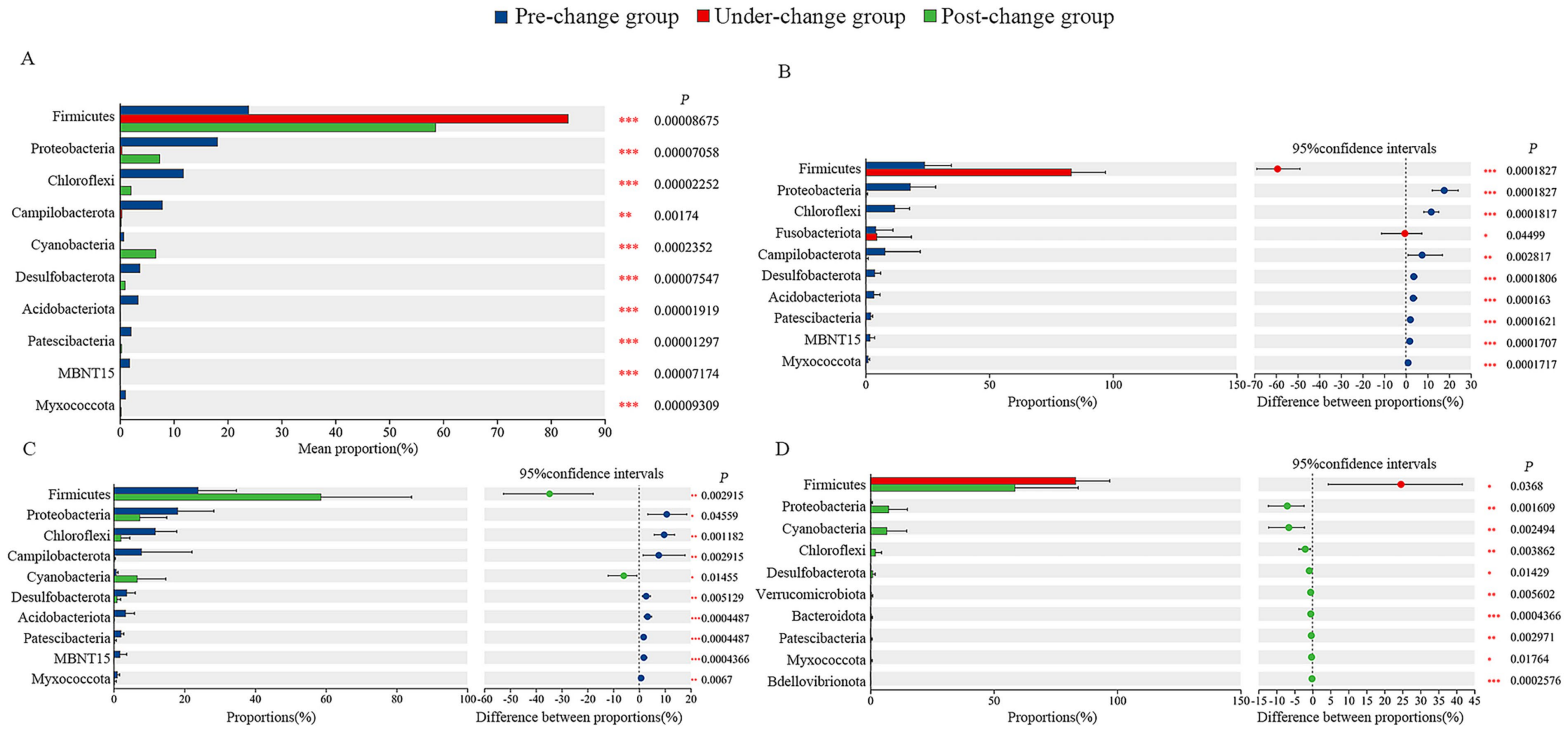
Comparison of microflora abundance at phylum level. The differential analysis of the dominant top 10 based on Kruskal-Wallis test among 3 years **(A)**; differential analysis of the top 10 bacterial phyla with abundance between pre-change and under-change groups **(B)**, pre-change and post-change groups **(C)**, under-change and post-change groups **(D)** based on Wilcoxon. * Indicated a significant difference (0.01 < *p* ≤ 0.05), ** indicated an extremely significant difference (0.001 < *p* ≤ 0.01), and ***indicated the existence of a super- significant difference (*p* ≤ 0.001).

To analyze differences in microbial abundance, the Kruskal-Wallis rank-sum test (for three-year comparisons) and Wilcoxon rank-sum test (for pairwise year comparisons) were applied to the top 10 phyla and top 15 genera. At the phylum level, significant interannual differences were observed: Campilobacterota showed extremely significant differences among the 3 years (0.001 < *p* ≤ 0.01), while 9 other phyla (including: Firmicutes, Proteobacteria, Chloroflexi) exhibited super-significant differences (*p* ≤ 0.001) (Figure 2A). For pairwise comparisons, the pre-change group had significantly higher abundances of key phyla relative to the under-change group: Campilobacterota was extremely significantly higher (0.001 < *p* ≤ 0.01), and 7 phyla (such as Proteobacteria, Chloroflexi, Desulfobacterota) were super-significantly higher (*p* ≤ 0.001) (Figure 2B). Specifically, compared with the post-change group, the pre-change group had a significantly higher abundance of Proteobacteria (0.01 < *p* ≤ 0.05), extremely higher abundances of 4 phyla (Chloroflexi, Campilobacterota, Desulfobacterota, Myxococcota; 0.001 < *p* ≤ 0.01), and super-significantly higher abundances of 3 phyla (Acidobacteriota, Patescibacteria, MBNT15; *p* ≤ 0.001) (Figure 2C). Additionally, the under-change group showed a significantly higher abundance of Firmicutes than the post-change group (0.01 < *p* ≤ 0.05) (Figure 2D).

**Figure 3:**
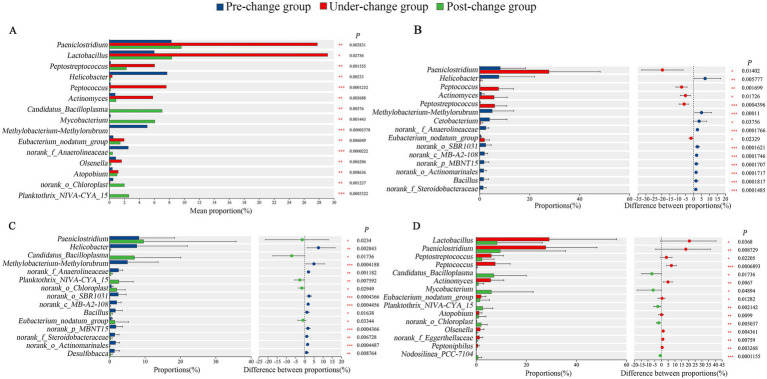
Comparison of microflora abundance at genus level. The differential analysis of the dominant top 15 based on Kruskal-Wallis test among 3 years **(A)**; differential analysis of the top 15 bacterial genus with abundance between pre-change and under-change groups **(B)**, pre-change and post-change groups **(C)**, under-change and post-change groups **(D)** based on Wilcoxon. * Indicated a significant difference (0.01 < *p* ≤ 0.05), ** indicated an extremely significant difference (0.001 < *p* ≤ 0.01), and *** indicated the existence of a super- significant difference (*p* ≤ 0.001).

At the genus level, significant interannual differences were also detected: *Lactobacillus* showed significant differences among the 3 years (0.01 < *p* ≤ 0.05), 10 genera (including *Paeniclostridium*, *Peptostreptococcus*, *Helicobacter*) exhibited extremely significant differences (0.001 < *p* ≤ 0.01), and 4 genera (*Peptococcus*, *Methylobacterium-Methylorubrum*, *norank_f__Anaerolineaceae*, *Planktothrix_NIVA-CYA_15*) showed super-significant differences (*p* ≤ 0.001) (Figure 3A). In pairwise comparisons, the pre-change group had a significantly higher abundance of *Cetobacterium* (0.01 < *p* ≤ 0.05) and an extremely higher abundance of *Helicobacter* (0.001 < *p* ≤ 0.01) compared with the under-change group, along with super-significantly higher abundances of 8 genera (including *Methylobacterium-Methylorubrum*, *norank_f__Anaerolineaceae*, *norank_o__SBR1031*; *p* ≤ 0.001) (Figure 3B). When compared with the post-change group, the pre-change group had a significantly higher abundance of *Bacillus* (0.01 < *p* ≤ 0.05), extremely higher abundances of 4 genera (*Helicobacter*, *norank_f__Anaerolineaceae*, *norank_f__Steroidobacteraceae*, *Desulfobacca*; 0.001 < *p* ≤ 0.01), and super-significantly higher abundances of 5 genera (such as *Methylobacterium-Methylorubrum*, *norank_o__SBR1031*, *norank_c__MB-A2-108*; *p* ≤ 0.001) (Figure 3C). In contrast, the under-change group had significantly higher abundances of 3 genera (*Lactobacillus*, *Peptostreptococcus*, *Eubacterium_nodatum_group*; 0.01 < *p* ≤ 0.05), extremely higher abundances of 6 genera (*Paeniclostridium*, *Actinomyces*, *Atopobium*, *Olsenella*, *norank_f__Eggerthellaceae*, *Peptoniphilus*; 0.001 < *p* ≤ 0.01), and a super-significantly higher abundance of *Peptococcus* (*p* ≤ 0.001) relative to the post-change group (Figure 3D).

#### 3.1.3 Alpha and beta diversity analysis

**Figure 4:**
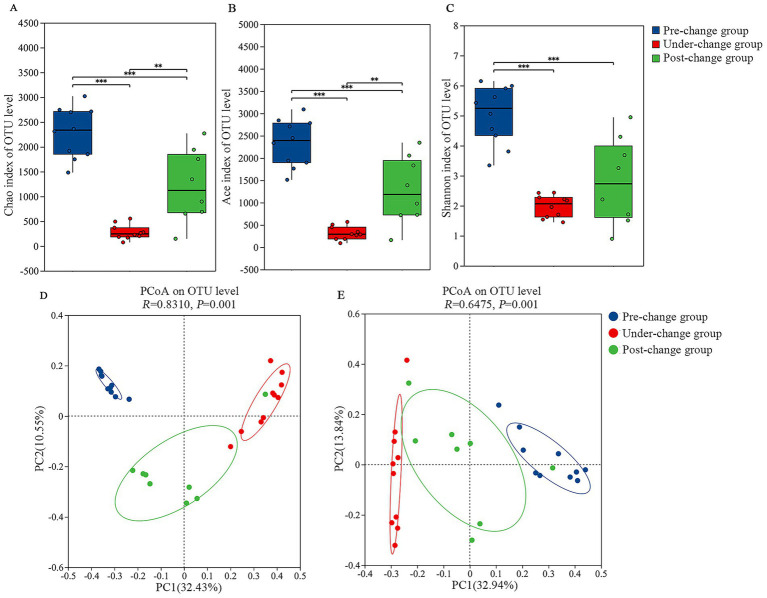
The comparison on the alpha and beta diversity across three groups. Differences in Chao **(A)**, Ace **(B)**, and Shannon **(C)** indices based on Kruskal-Wallis; Beta diversity analysis of the gut microbiota among 3 years based on unweighted Unifrac distances **(D)** and weighted Unifrac distances **(E)**.

Microbial richness was quantified using Ace and Chao indices, while diversity was assessed via Shannon index. Kruskal-Wallis tests revealed significant differences across all indices (Figures 4A–C). The pre-change group exhibited super significant differences than both under-change group and post-change group phases (*p* ≤ 0.001). Furthermore, under-change group indices were extremely significantly lower than post-change group values (Ace/Chao: 0.001 < *p* ≤ 0.01), indicating maximal richness and diversity during pre-change group and minimal during under-change group.

Principal coordinates analysis (PCoA) based on Unweighted/Weighted UniFrac metrics demonstrated distinct clustering patterns: pre-change group and under-change group communities showed greater compositional similarity, while post-change group samples displayed increased dispersion (Figures 4D,E). Both distance metrics confirmed significant community structure differences among groups (Unweighted UniFrac: *R* = 0.8310, *p* = 0.001; Weighted UniFrac: *R* = 0.6475, *p* = 0.001).

#### 3.1.4 Function and pathogenicity prediction

**Figure 5:**
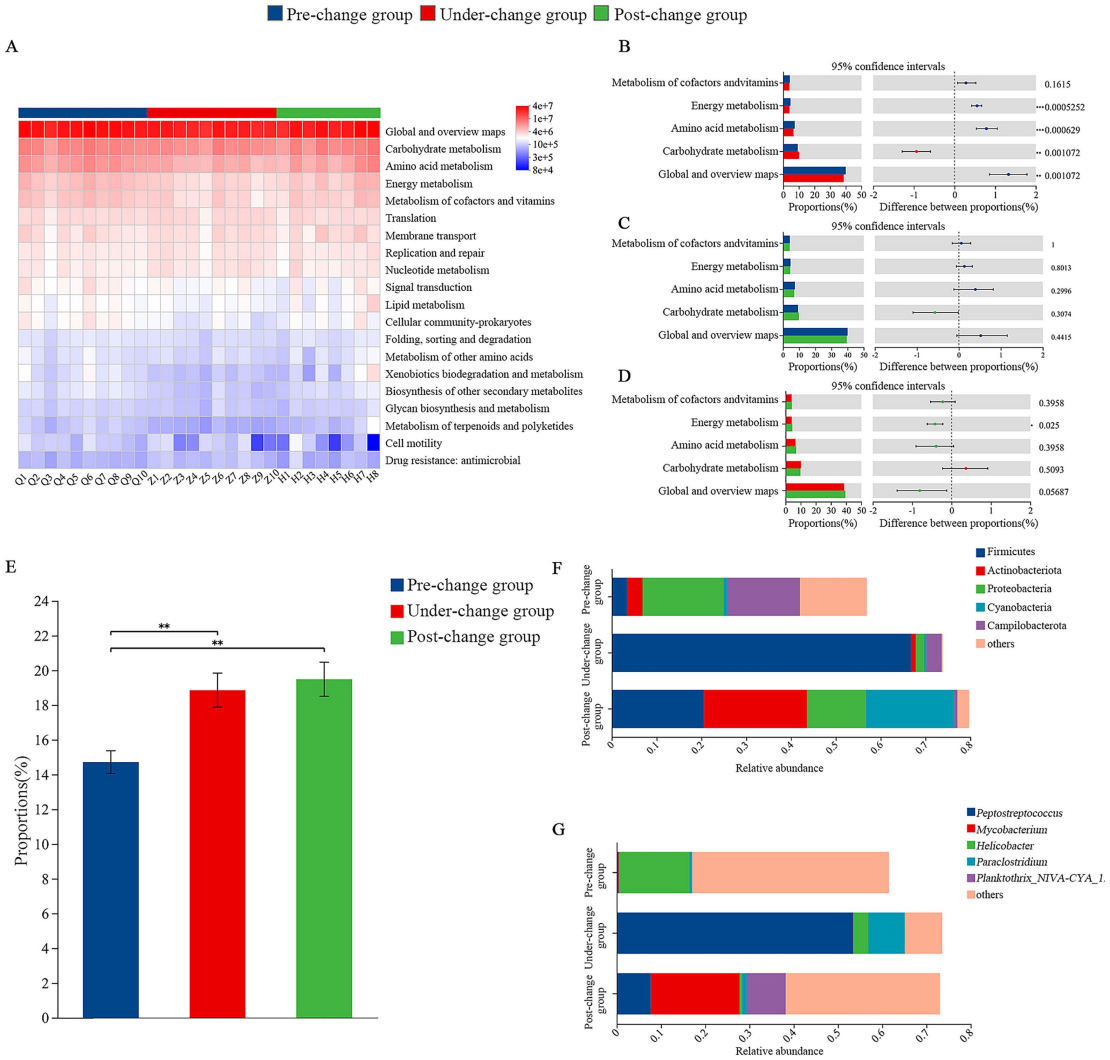
The functional prediction based on high-throughput sequencing. Functional prediction of gut microbiota among three groups **(A)**; the differential analysis of the top five abundant functions based on PICRUST 2.0 between pre-change and under-change groups **(B)**, pre-change and post-change groups **(C)**, under-change and post-change groups **(D)**; prediction of potential pathogenicity **(E)** of gut microbiota in *C. boyciana* and analysis of contribution of phyla **(F)** and genera **(G)**. * Indicated a significant difference (0.01 < *p* ≤ 0.05), ** indicated an extremely significant difference (0.001 < *p* ≤ 0.01), and *** indicated the existence of a super-significant difference (*p* ≤ 0.001).

This study used PICRUST 2.0 to predict the functions of the gut microbiota in oriental storks and conducted differential analysis. At the secondary functional classification level, 46 functional clusters were obtained, with high-abundance functions including global and overview maps, carbohydrate metabolism, amino acid metabolism, energy metabolism, and metabolism of cofactors and vitamins (Figure 5A). Among them, 13 functional clusters were not statistically significant. Differential analysis was performed on the top five abundant functions. There were extremely significant differences in the abundances of global and overview maps and carbohydrate metabolism functions between the pre-change and under-change groups (0.001 < *p* ≤ 0.01) (Figure 5B), and super significant differences in the abundances of amino acid metabolism and energy metabolism functions (*p* ≤ 0.001). There were significant differences in the abundance of energy metabolism function between the under-change and post-change groups (0.01 < *p* ≤ 0.05) (Figure 5D).

This study used BugBase to predict the potential pathogenicity of the gut microbiota in oriental storks and analyzed the gut microbiota contribution. As shown in Figure 5, the potential pathogenicity was lowest in the pre-change group and extremely significantly lower than that in the under-change and post-change groups (0.001 < *p* ≤ 0.01) (Figure 5E). The top five phyla and genera contributing to potential pathogenicity were analyzed. At the phylum level, Proteobacteria (18.12%) and Campilobacterota (16.10%) had the highest relative abundance of pathogens in the pre-change group; Firmicutes (66.78%) had the highest relative abundance of pathogens in the under-change group; Actinobacteria (22.98%) and Firmicutes (20.57%) had the highest relative abundance of pathogens in the post-change group (Figure 5F). At the genus level, *Helicobacter* (15.88%) had the highest relative abundance of pathogens in the pre-change group; *Peptostreptococcus* (53.41%) and *Paraclostridium* (8.25%) had the highest relative abundance of pathogens in the under-change group; *Mycobacterium* (20.06%) and *Planktothrix_NIVA-CYA_15* (8.80%) had the highest relative abundance of pathogens in the post-change group (Figure 5G).

### 3.2 Comparison of microscopic examination results

This study conducted microscopic tissue analysis on the fecal samples of oriental storks. Referring to the microscopic structure of plants in Tianjin Qilihai Wetland, plants in the photographs were identified and classified (Fecal samples from the post-change group were not subjected to microscopic examination due to insufficient sample volume) As shown in Table 1, a total of 8 families, 10 genera, and 10 species of plants were identified. Oriental storks consumed more plant species (9 species) in the under-change group than in the pre-change group (8 species). There were a total of 5 families, 7 genera and 7 species of plants in the two groups. They, respectively, included *Phragmites australis, Echinochloa crusgalli, Digitaria sanguinalis, Cynanchum chinense, Myriophyllum spicatum, Bolboschoenus yagara*, and *Pharbitis nil*. In the pre-change group, the proportions of plants except *P. australis* were all lower than those in the under-change group. In the pre-change group, there was 1 family, 1 genus and 1 species, namely *Abutilon theophrasti*. In the under-change group, there were 2 families, 2 genera and 2 species, namely *Suaeda glauca* and *Nelumbo nucifera*.

**Table 1:** Collation of plants examined by microscopy from oriental storks living in Tianjin Qilihai Wetland.

Family	Genera	Species	Proportion (%)	Proportion (%)
			Pre-change group	Under-change group
Poaceae	*Phragmites*	*Phragmites australis*	14.25	9.52
	*Echinochloa*	*Echinochloa crusgalli*	6.36	9.52
	*Digitaria*	*Digitaria sanguinalis*	1.53	4.76
Malvaceae	*Abutilon*	*Abutilon theophrasti*	16.54	0
Asclepiadaceae	*Cynanchum*	*Cynanchum chinense*	5.09	16.67
Haloragidaceae	*Myriophyllum*	*Myriophyllum spicatum*	1.02	11.90
Cyperaceae	*Bolboschoenus*	*Bolboschoenus yagara*	1.27	8.33
Convolvulaceae	*Pharbitis*	*Pharbitis nil*	4.33	5.95
Chenopodiaceae	*Suaeda*	*Suaeda glauca*	0	3.57
Nymphaeaceae	*Nelumbo*	*Nelumbo nucifera*	0	8.33
Unidentified plant			49.62	21.43

### 3.3 Correlation analysis between gut microbiota and plant diet

**Figure 6:**
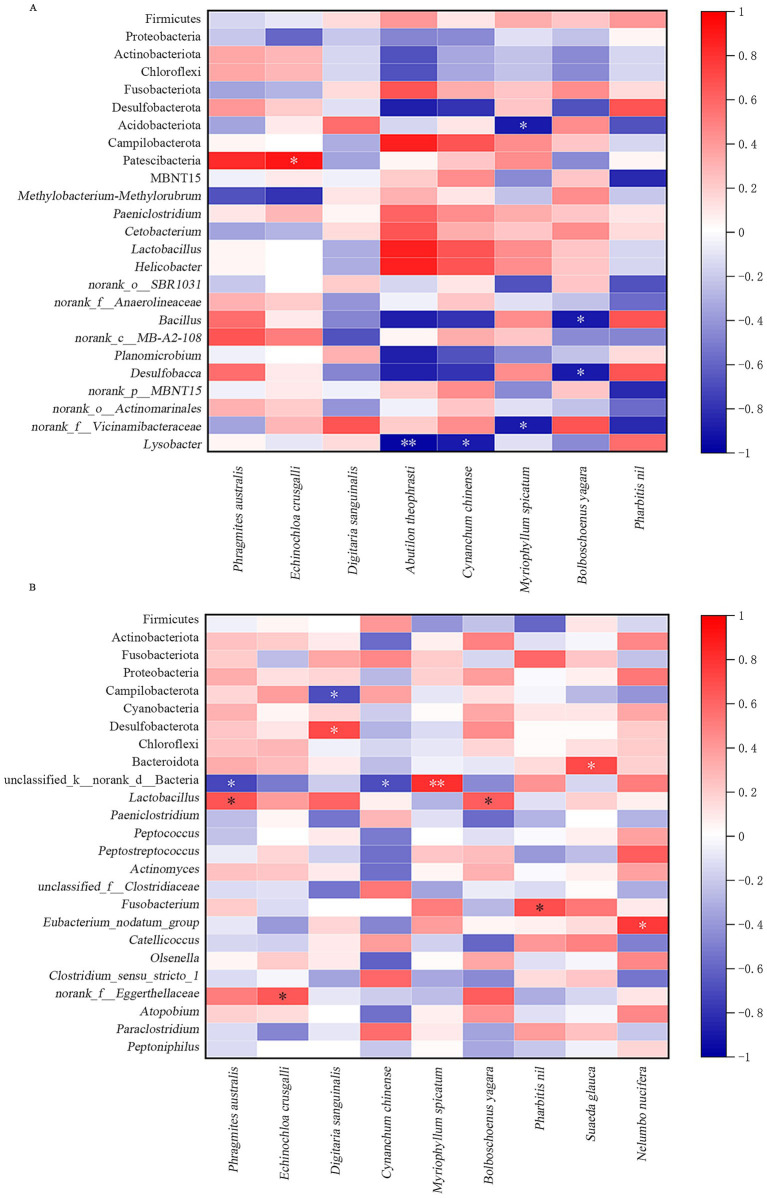
The relationship between diet composition and gut microbiota. The heatmap of the correlation between the relative density of plants and the abundance of gut microbiota from pre-change group **(A)** and under-change group **(B)**.

This study conducted correlation analysis between the relative density (*RD*) of consumed plants and the abundances of the top 10 phyla and top 15 genera of gut microbiota in oriental storks during the pre-change and under-change groups. The Spearman correlation results were shown in Figure 6. The results indicated that the abundance of gut microbiota in oriental storks were related to their plant diet. In the pre-change group, Acidobacteriota and *norank_f__Vicinamibacteraceae* were significantly negatively correlated with *M. spicatum* (0.01 < *p* ≤ 0.05); *Bacillus* and *Desulfobacca* were significantly negatively correlated with *B. yagara* (0.01 < *p* ≤ 0.05); *Lysobacter* was significantly negatively correlated with *C. chinense* (0.01 < *p* ≤ 0.05); Patescibacteria was significantly positively correlated with *E. crusgalli* (0.01 < *p* ≤ 0.05); *Lysobacter* was extremely significantly negatively correlated with *A. theophrasti* (0.001 < *p* ≤ 0.01). In the under-change group, Campilobacterota was significantly negatively correlated with *D. sanguinalis* (0.01 < *p* ≤ 0.05); unclassified_k__norank_d__Bacteria was significantly negatively correlated with both *P. australis* and *C. chinense* (0.01 < *p* ≤ 0.05); Desulfobacterota was significantly positively correlated with *D. sanguinalis* (0.01 < *p* ≤ 0.05); Bacteroidota was significantly positively correlated with *S. glauca* (0.01 < *p* ≤ 0.05); *Lactobacillus* was significantly positively correlated with *P. australis* and *B. yagara* (0.01 < *p* ≤ 0.05); *Fusobacterium* was significantly positively correlated with *P. nil* (0.01 < *p* ≤ 0.05); *Eubacterium_nodatum_group* was significantly positively correlated with *N. nucifera* (0.01 < *p* ≤ 0.05); *norank_f__Eggerthellaceae* was significantly positively correlated with *E. crusgalli* (0.01 < *p* ≤ 0.05); unclassified_k__norank_d__Bacteria was extremely significantly positively correlated with *M. spicatum* (0.001 < *p* ≤ 0.01).

## 4 Discussion

### 4.1 Gut microbiota composition

By analyzing the fecal microbial composition of oriental storks at different stages across three consecutive years consistently, the results demonstrated that at the phylum level, the top three dominant phyla included Firmicutes and Actinobacteria, consistent with our previous findings (20). Firmicutes is prevalent among numerous migratory bird species including black-necked cranes (*Grus nigricollis*), mallards (*Anas platyrhynchos*), and bean geese (*Anser fabalis*) (41–43). Firmicutes ferments carbohydrates, polysaccharides, and lipids (44, 45), facilitating host nutrient absorption and energy acquisition. Critically, Firmicutes-derived short-chain fatty acids (SCFAs) regulate host metabolism and mitigate intestinal disorders (46).

During habitat micromodification, Firmicutes abundance exhibited significant dynamic changes. It was substantially higher in the under-change group than in pre-change group and post-change group, while remaining elevated in post-change group compared to pre-change group. This pattern coincided with water replenishment activities at Tianjin Qilihai Wetland, which reduced water levels and suspended fish stocking. As the principal energy source for oriental storks, reduced availability of fish resources may decrease energy intake. PICRUSt2-based functional prediction revealed significantly lower abundance of microbial energy metabolic pathways during the under-change group versus pre-change, with a recovery tendency in post-change group. These functional shifts are temporally aligned with dietary perturbation, suggesting potential host energy adaptation challenges. As facultative piscivores shifting to plant-based foods (e.g., reed rhizomes, aquatic seeds) during fish scarcity (47), storks likely increased consumption of high-fiber vegetation during under-change group, potentially driving Firmicutes proliferation to enhance plant polysaccharide digestion.

Actinobacteria, also a dominant phylum, facilitates the degradation of chitin and cellulose and helps maintain gut-immune homeostasis (48–50). Notably, the dominant phyla in pre-change group were characterized by Firmicutes, Proteobacteria, and Actinobacteria, whereas under-change and post-change group featured Firmicutes, Actinobacteria, and Fusobacteriota. The abundance of Fusobacteriota was significantly higher in under-change group than in pre-change group.

Fusobacteria, which are widely distributed in nature, include species that produce butyrate to aid the host in fat absorption and form biofilms to prevent pathogen colonization and invasion (51, 52). According to previous studies, Fusobacteria is dominant bacteria in *Anser anser* (53), *Calypte anna* (54), *Gyps himalayensis* (55) and other birds. Some studies found that Fusobacteria is harmful to species, such as *Fusobacterium nucleatum*, which is associated with enteritis (56). Stokowa-Sołtys et al. (57) found that *F. nucleatum* can produce butyrate and act on the immune system to prevent the invasion of pathogens. This study speculates that the increased abundance of Fusobacteria may reflect the enrichment of potential pathogenic bacteria caused by changes in the wetland environment, or the physiologically adaptive adjustment of the host immune system.

At the genus level, *Paeniclostridium* and *Lactobacillus* persisted as dominant taxa across all phases. *Paeniclostridium*, a known pathobiont linked to immune dysregulation (58), peaked during under-change group. This coincided with increased abundances of other potential pathogens (e.g., *Actinomyces*) (59). Compensatory increases in beneficial bacteria like *Lactobacillus* [crucial for gut barrier repair; (60, 61)] was observed during this phase, with its abundance significantly higher in under-change group than post-change group. Furthermore, enteritis-associated genera *Peptostreptococcus* and *Peptococcus* (62, 63) peaked in under-change group. Studies confirm that identical pathogenic bacteria exhibit variable pathogenicity and symptom presentation among individual hosts (64). Consequently, establishing clinical relevance requires correlating microbial data with species-specific manifestations. Currently, the pathogenicity of the genera identified in this study remains unclear for oriental storks. While our findings may not directly reflect this species’ clinical health status, they lay essential groundwork for future investigations into stork-specific pathogen dynamics.

Collectively, these microbial shifts may indicate compromised host health during active modification. The post-change group showed reduced pathobiont abundances and increased beneficial taxa like *Lactobacillus* and *Eubacterium_nodatum_group* which could enhance SCFA production compared to pre-change group (61, 65). This underscores that habitat micromodification transiently threatens oriental storks, health, necessitating future management protocols that minimize disturbance during critical life-history stages (e.g., migration, breeding) and ensure adequate food provisioning.

Declining water levels in the species’ habitat will impact the diversity and availability of its food resources (66). The results of this study indicated that the diversity of the species’ gut microbiota was related to environmental modifications. In 2023, the water level in Tianjin Qilihai Wetland was the lowest, leading to a decrease in the number of aquatic plants and a scarcity of food resources. The number of fish and benthic organisms available for the oriental stork to feed on also decreased (67). The reduction in food resources increases competition among species with similar dietary preferences, makes it more difficult to hunt fish, and increases energy consumption. Consequently, the oriental stork may shift to consuming more plants, which in turn alters the diversity and functionality of their gut microbiota. For example, compared to the pre-change group, the number of microbial communities such as *Cetobacterium*, which are obtained from fish (68), decreased in under-change group. Additionally, the abundance of gut microbiota related to energy metabolism was super significantly lower in under-change group compared to the pre-change group and was notably lower than post-change group. This further suggests that fewer fish were available for the oriental stork in the under-change group. In the post-change group, the water level in the wetland recovered, leading to an increase in the number of aquatic plants. During the post-change group, the abundance of *Candidatus_Bacilloplasma* was significantly higher than in the pre-change group and under-change group. Xiao et al. (68) demonstrated that food-derived microbiota shape host gut communities. Crustaceans, including crabs and shrimps in the oriental stork’s diet, harbor high abundances of *Candidatus_Bacilloplasma* in their enteric microbiota (69, 70). We therefore propose that increased *Candidatus_Bacilloplasma* abundance during the post-change group may reflect heightened crustacean availability in the environment.

*Ureaplasma* can hydrolyze urea and is commonly present in avian intestines [e.g., barn swallow *Hirundo rustica*, (71); great tit *P. major*, (72)]. Bodawatta et al. (27) found that under high-protein diets, the abundance of *Ureaplasma* increased in the intestines of great tits (*P. major*). Zeber-Lubecka et al. (73) indicated that under high-fat conditions, the abundance of *Ureaplasma* increased in mouse intestines. The increased abundance of *Ureaplasma* during the post-change group may result from heightened protein and fat intake due to enriched wetland food resources (e.g., increased consumption of fish and crustaceans), but its association with specific diets requires further study.

Prior studies indicate that groups within a shared environment develop convergent gut microbiota due to similar living conditions, comparable diets, and social behaviors such as mutual preening (74, 75). Our results in pre-change and under-change groups align with this pattern. However, microbiota composition diverged in post-change group, evidenced by greater dispersion among samples and increased inter-individual variability. Although factors like sex, age, and dietary preferences are known to drive microbial differences (76, 77), logistical constraints prevented verification of individual traits (e.g., foraging choices, demographics) during this phase. We propose that physiological status and dietary selection jointly contributed to these outcomes, though mechanistic details require further investigation.

### 4.2 Dietary composition

Microscopic analysis of indigestible fragments (microhistology) has long been used to monitor diets of wildlife, and a total of 10 plant species (8 families, 10 genera) were identified from the fecal samples of oriental storks. *A. theophrasti* was exclusive to pre-change group, while *N. nucifera* and *S. glauca* appeared only during under-change group. Seven species persisted across phases, including terrestrial plants (*E. crusgalli*, *D. sanguinalis, C. chinense*, *P. nil*) and aquatic plants (*P. australis, M. spicatum, B. yagara*). The analysis of the dietary habits of oriental storks feces revealed the presence of common plant species in Tianjin coastal wetlands, such as *Phragmites australis, Echinochloa crusgalli, Digitaria sanguinalis.*

Water-level declines increased light penetration, boosting *M. spicatum* biomass and protein content (78), explaining its higher dietary proportion in under-change group (11.9% vs. 1.02% pre-change group). Concurrently, Acidobacteria abundance plummeted. As *M. spicatum* contains abundant tannins (79), known to suppress Bacteroides and elevate Clostridiales in poultry (80) and reduce Acidobacteria in natural systems (81), we propose tannin-mediated inhibition as a plausible mechanism for this inverse correlation.

Water-level reductions likely exposed lotus (*N. nucifera*) rhizomes, facilitating storks access. Its high carbohydrate content (82) coincided with increased *Eubacterium_nodatum_group*, a known carbohydrate-fermenting SCFA producer (83, 84), suggesting microbial adjustments to dietary shifts. The 2023 environmental micromodification at Tianjin Qilihai Wetland involved both water level reduction and levee reinforcement. These interventions directly altered the composition of subaquatic soils and the distribution pattern of aquatic vegetation. Consequently, the observed changes in avian gut microbiota should be interpreted as a collective outcome of the integrated habitat micromodification efforts. While water level decline served as the most conspicuous driver which directly diminishing primary food sources such as fish, ancillary modifications, particularly levee construction, imposed additional selective pressures on microbial communities. Synthetically, the habitat micromodification itself likely acted as the principal catalyst for microbial community restructuring, whereas hydrological alterations primarily influenced microbiota indirectly through trophic resource mediation.

Although *P. australis* consumption decreased in under-change group (9.52% vs. 14.25% pre-change group), *Lactobacillus* abundance increased paradoxically. As cellulose intake elevates *Lactobacillus* in mammals (85, 86), we attribute this to higher overall plant consumption (increasing total cellulose), and kaempferol (a flavonoid in post-change group) [*S. glauca*; (87)] which consistently enriches *Lactobacillus* across vertebrates (88, 89). Increased under-change group consumption of *B. yagara* (8.33% vs. 1.27% pre-change group), containing immune-modulating resveratrol and betulin (90), further supported *Lactobacillus* enrichment, as resveratrol ameliorates gut dysbiosis in birds (91), rodents (92), and swine (93). Comparative analyses revealed that declining water levels triggered increased consumption of fibrous plant matter in *Grus grus*, accompanied by rising Lactobacillus abundance in their gut microbiota (94). This microbial adaptation pattern mirrors our findings in oriental storks during under-change group, demonstrating how wading birds employ functional microbiome restructuring to cope with nutritional changes during habitat fluctuations. When fish meal was partially replaced by *Tenebrio molitor* meal in the diet of defatted yellow mealworm (*Larimichthys crocea*), a significant increase in intestinal *Lactobacillus* abundance was observed (95). The reduction in fish-derived protein during the under-change group may partially explain the elevated *Lactobacillus* levels. Therefore, we propose that the increased *Lactobacillus* abundance in our study could be collectively driven by both plant-based and animal-based dietary components. Kaempferol also drove Bacteroidota enrichment during under-change group (96, 97), absent in pre-change group when *S. glauca* was unconsumed. These results indicate that environmental micromodification affected the type and quantity of available food resources, which in turn influenced the foraging behavior and gut microbiota structure of oriental storks. This demonstrated the oriental stork’s dual adaptive strategies: physiological adjustments and behavioral plasticity in response to environmental changes. From a management perspective, it is recommended that engineering activities avoid migration and breeding seasons, that water levels be maintained to support aquatic prey populations, and that food supplementation be provided during disturbance periods to reduce foraging costs. This study provides a scientific basis for the restoration of degraded habitats of endangered species by elucidating the interactions among diet, habitat, and gut microbiota. Prior to modification, thorough investigation into species-specific ecological niche requirements should be conducted to minimize ecological disruption. During modification, key prey resources should be enhanced to counteract microbiota dysbiosis caused by food web fragmentation. Post-modification, continuous monitoring of microbial diversity and habitat recovery indicators is essential to dynamically adjust management strategies, thereby promoting functional habitat recovery and ensuring suitable conditions for reproduction, roosting, and survival.

While oriental storks primarily consume animal-based foods, the link between plant intake and gut microbiota should not be overlooked. As documented in Marabou storks, a dietary shift toward omnivory due to habitat alteration induced significant microbiota restructuring (98). This demonstrates that food consumption influences microbial communities even in predominantly carnivorous species. Consequently, gut microbiota composition likely represents a composite outcome of both animal and plant dietary inputs. However, the inherent limitations of microscopic analysis, such as its inability to identify specific animal species and inherent quantification biases, prevent comprehensive animal dietary data collection in this study. This gap may confound correlations between plant intake and microbiota composition.

During the post-change group, microscopic analysis could not be performed on some fecal samples due to limited sample volume. Consequently, dietary data only cover 2022–2023, while microbial data span 2022–2024. This discrepancy may affect direct support for dietary recovery conclusions. However, it’s noteworthy that core phyla like Firmicutes and Proteobacteria showed abundance shifts during the under-change group, which then approached pre-change group levels in post-change group. Similarly, key genera such as *Paeniclostridium* and *Lactobacillus* exhibited parallel trends. These microbial patterns may indirectly reflect a restoration of dietary preferences toward pre-change group conditions.

## 5 Conclusion

In summary, this study compared gut microbiota composition of oriental storks across pre-, under-, and post-change groups, alongside dietary composition during pre-change and under-change group. The research revealed that wetland water level decline during environmental micromodification reduced diversity and abundance of food resources, consequently affecting dietary selection and gut microbiota structure. Increased abundances of potential pathogens (e.g., *Actinomyces*, *Paeniclostridium*) during micromodification indicated health risks posed by habitat disturbance. This conclusion represents an extrapolation based on existing literature and BugBase bioinformatic analysis. It may not fully capture direct clinical correlations with the species’ health status. Post-change group witnessed decreased pathogenic bacteria abundances, while beneficial bacteria (e.g., *Lactobacillus*, *Eubacterium_nodatum_group*) increased compared to pre-change group. Future environmental modification projects must therefore prioritize comprehensive assessment of health impacts on target species. The absence of animal dietary data may affect diet-microbiota relationship interpretations. Due to the inherent limitations of 16S rRNA high-throughput sequencing, our current dataset cannot fully elucidate the underlying mechanisms linking dietary habits with gut microbiota composition. Future investigations will employ an integrated approach combining metagenomic sequencing and DNA metabarcoding techniques to comprehensively characterize the dietary composition of oriental storks and its functional correlations with intestinal microbial communities. Nevertheless, this study provides the first evidence linking plant-derived food intake with microbiota characteristics in oriental storks, establishing a foundational reference for subsequent diet-microbiota investigations.

## Data Availability

All of the raw reads are now available at the NCBI database under PRJNA1007256 (SRX21506339 to SRX21506346 and SRX21506349 to SRX21506350) and PRJNA1284011 (SRX29462429 to SRX29462446).

## Footnotes

1
http://www.cbcb.umd.edu/software/flash
