# Supplementary material for: Dietary shifts and gut microbiota plasticity correlates of habitat micromodification in wild oriental storks: implications for conservation physiology
Source: Front Vet Sci. 2026 Feb 10;13:1769005. doi: 10.3389/fvets.2026.1769005 (PMC12929150; doi:10.3389/fvets.2026.1769005)
Supplement: Supplementary file 1 [file Data_Sheet_1.docx]

Supplementary Material

# Supplementary Figures

##
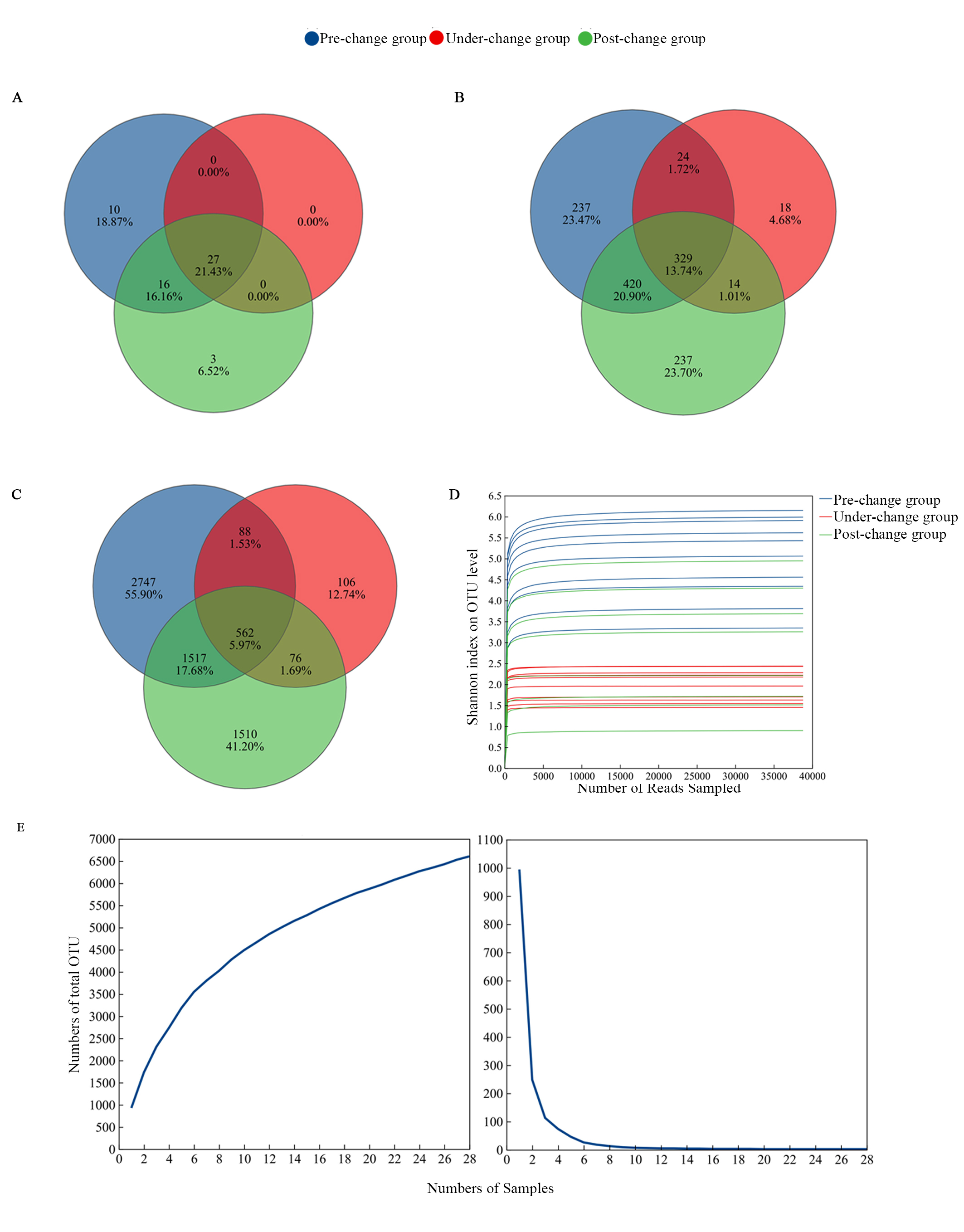


## Supplementary Figure 1. Venn analysis and rarefaction curve of high-throughput sequencing

## Venn diagram of three groups at phylum-level (A), genus-level (B) and OTU level (C); Rarefaction curve of three groups(D); Pan and Core (E) graph of gut microbiota.


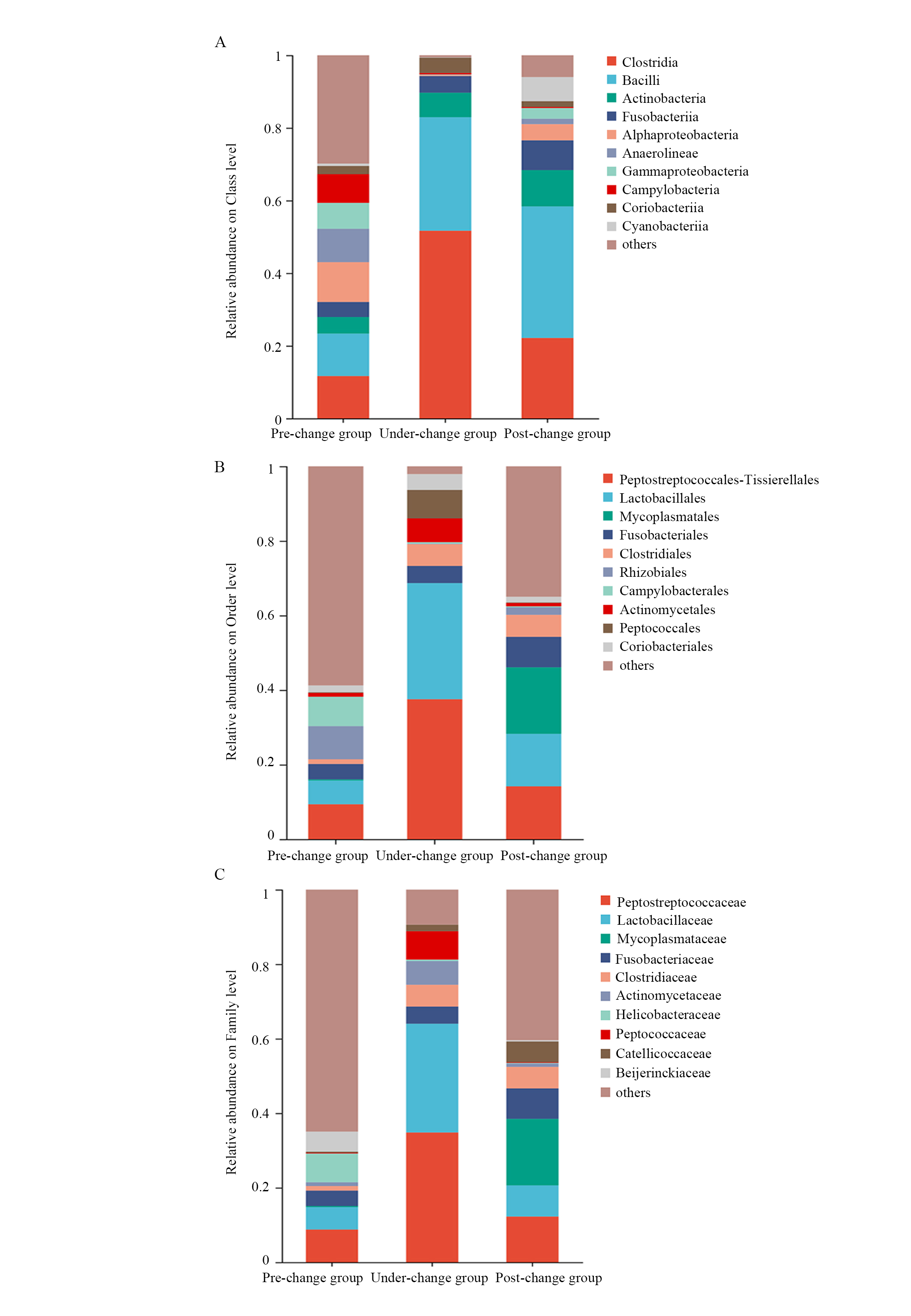


**Supplementary Figure 2.** Microbial structure of all fecal samples at class, order and family levels

(A) Bar-plots showing the abundance and distribution of the 10 most abundant class. (B) Bar-plots showing the abundance and distribution of the 10 most abundant order. (C) Bar-plots showing the abundance and distribution of the 10 most abundant family.


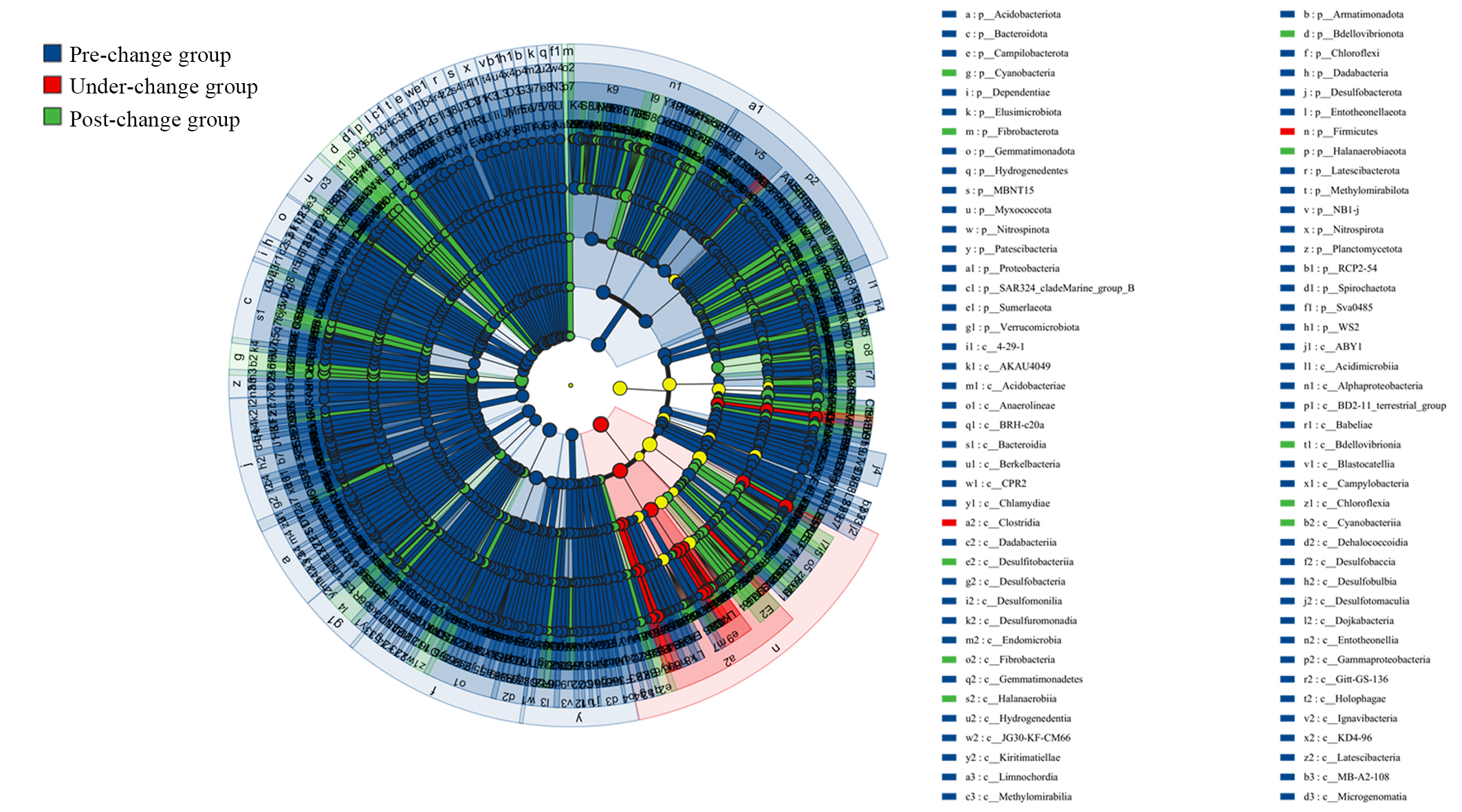


**Supplementary Figure 3.** LEfSe analysis of gut microbiota

LEfSe analysis based on characterizing discriminative features of OTUs.

# Supplementary Tables

**Table S1.**  Information of oriental storks from Tianjin Qilihai Wetland in this study.

| Groups | Sampling Time | Number | Sample Collection Sites |
| --- | --- | --- | --- |
| Pre-change group | 2022.11 | 10 | Tianjin Qilihai Wetland |
| Under-change group | 2023.11 | 10 | Tianjin Qilihai Wetland |
| Post-change group | 2024.11 | 8 | Tianjin Qilihai Wetland |

**Table S2.** The average relative abundances of the 5 most abundant phylum and 5 genus in each sample group.

| Groups | Phylum (%) | Genus (%) |
| --- | --- | --- |
| Pre-change group | Firmicutes (23.89%)  Proteobacteria (18.11%)  Actinobacteriota (14.87%)  Chloroflexi (11.79%)  Campilobacterota (7.89%) | *Paeniclostridium* (8.29%)  *Helicobacter* (7.72%)  *Lactobacillus* (6.00%)  *Methylobacterium-Methylorubrum* (5.07%)  *Cetobacterium* (4.00%) |
| Under-change group | Firmicutes (83.19%)  Actinobacteriota (11.08%)  Fusobacteriota (4.60%)  Proteobacteria (0.40%)  Campilobacterota (0.39%) | *Lactobacillus* (29.17%)  *Paeniclostridium* (27.79%)  *Peptococcu*s (7.61%)  *Peptostreptococcus* (6.05%)  *Actinomyces* (5.82%) |
| Post-change group | Firmicutes (58.60%)  Actinobacteriota (13.54%)  Fusobacteriota (8.18%)  Proteobacteria (7.40%)  Cyanobacteria (6.67%) | *Ureaplasma* (10.02%)  *Paeniclostridium* (9.62%)  *Lactobacillus* (8.35%)  *Candidatus_Bacilloplasma* (7.05%)  *Mycobacterium* (6.08%) |
